# Association of C-Reactive Protein and Outcome in Patients With Community-Acquired Pneumonia

**DOI:** 10.1186/2197-425X-3-S1-A523

**Published:** 2015-10-01

**Authors:** JK Park, J Lee, K Kim, YH Jo, JH Lee, J Kim, SM Lee, IA Song

**Affiliations:** Seoul National University Bundang Hospital, Seongnam-si, Republic of Korea

## Introduction

Physicians tend to think that high C-reactive protein (CRP) and increase of CRP are associated with bad outcome in infectious disease. However there were little evidences about clinical interpretation of C-reactive protein (CRP) value in patient with community-acquired pneumonia (CAP). We investigated association of CRP values and 30-day mortality in patients with community-acquired pneumonia who were admitted to emergency department (ED).

## Objectives

The objective of this study was to investigate whether high CRP and increase of CRP were associated with 30 day mortality in patients with CAP.

## Methods

The patients who were diagnosed with CAP in ED and hospitalized from January 2009 to January 2015 were enrolled. We investigated that CRP during 3 days after ED admission and 30-day mortality. the dynamic changes of CRP values per hour (%/hour) were calculated. After drawing graphs of CRP values, area under the curve (AUC) was calculated to measure mortality predictability. to investigate mortality predictability of CRP and dynamic change of CRP, univariable analysis result was adjusted by adjusted by pneumonia severity index[1].

## Results

A total of 1194 patients were analyzed retrospectively. Overall mortality was 14.9%. Median time (interquartile range) of day 2 and day 3-CRP values from admission was 18.2 hours (14.6-21.6) and 42.5 hours (38.8-45.9). High CRP values during admission period were associated with 30-day mortality; initial CRP, adjusted odds ratio (AOR) 1.02 95% confidence interval (CI) 1.00-1.04; Day-2 CRP, AOR 1.03 95% CI 1.01-1.05; Day-3 CRP, AOR 1.05 95% CI 1.02-1.09. Dynamic changes of each CRP values and AUCs of CRP values were not associated with 30-day mortality.

## Conclusions

High CRP was independent risk factor to predict 30-day mortality in patients with CAP. Dynamic changes of CRP values were not associated with 30-day mortality.
